# SOCIAL DETERMINANTS OF HEALTH DRIVE EMERGENCY ROOM AND HOSPITAL USE BY DUAL ELIGIBLE AND DISABLED PENNSYLVANIANS

**DOI:** 10.1093/geroni/igz038.3495

**Published:** 2019-11-08

**Authors:** Sih-Ting Cai, Howard Degenholtz, Hayley Germack

**Affiliations:** University of Pittsburgh, Pittsburgh, Pennsylvania, United States

## Abstract

The study examined correlates and consequences of social determinants of health risk factors (SDoH) among dual eligible aged and disabled individuals; Pennsylvania is transitioning this population into a managed care plan with responsibility for care coordination and incentives to prevent hospitalization and nursing home placement. Medicaid and Medicare claims were used to identify people with SDoH based on ICD-10 codes in 2016 in four domains: economic insecurity, life stressors, physical dependence, and potential health hazards. Of 281,918 people, 38.6% had one or more SDoH. Among people with severe mental illnesses (SMI; schizophrenia, psychosis, major depressive disorder, or bipolar disorder), the prevalence of SDoH was 57.9%. Of people with one or more SDoH, 42% visited the ED, compared to only 32% of people with no SDoH. Economic insecurity (OR 1.68; CI 1.59-1.78), life stressors (OR 1.39; CI 1.29-1.48), physical dependence, (OR 2.01; CI 1.97-2.06), and potential health hazards (OR 1.52; CI 1.47-1.56) were independently associated with risk of hospitalization, controlling for age, gender, race, SMI, chronic conditions and disability. The introduction of diagnosis codes for SDoH under ICD-10 has facilitated identifying individuals with deficits that might increase health care use above and beyond their underlying health status. Although the prevalence of these risk factors as captured in diagnosis data is likely an underestimate, the strong association with subsequent ED use and hospitalization lends credence to these indicators. Medicare and Medicaid claims data can be used to identify people with SDoH and target interventions to prevent downstream health services use.

